# Successful sublingual immunotherapy for severe egg allergy in children: a case report

**DOI:** 10.1186/s13223-020-00506-1

**Published:** 2021-01-06

**Authors:** Nagatoshi Sagara, Satoshi Fujita, Ryohei Suzuki, Akiko Aota, Kenichi Akashi, Toshio Katsunuma

**Affiliations:** Department of Pediatrics, Daisan Hospital, The Jikei University School of Medicine, 4-11-1, Izumihoncho, Komae-shi, Tokyo 201-8601 Japan

**Keywords:** Case report, Egg, Food allergy, Pediatrics, Sublingual immunotherapy

## Abstract

**Background:**

Egg allergy is one of the most common food allergies in children. To date, oral immunotherapy (OIT) has been considered as a promising treatment option for egg allergy. However, safety issues remain concerning severe adverse events requiring epinephrine injection. Hence, establishing a safer method to treat egg allergy would be beneficial. We report here two children with egg allergy who were safely treated with sublingual immunotherapy (SLIT) before transitioning to OIT.

**Case presentation:**

Patient 1 was a 7-year-old girl and Patient 2 was a 5-year-old girl. Although OIT for egg had been attempted in both patients, severe anaphylactic symptoms were induced by ingesting only 0.1 g of heated whole egg. Therefore, SLIT was conducted with aqueous suspensions consisting of water and heated whole egg powder. Suspensions were administered sublingually, kept in the mouth for 2 min, and spat out immediately thereafter. SLIT was continued for 7 months for Patient 1 and 8 months for Patient 2 due to the exploratory character of the study. Afterwards, the patients successfully transferred to low-dose OIT with 1 g of heated whole egg (≒170 mg of egg protein) daily, and are continuing the therapy as of June 2020. As for adverse reactions, Patient 1 expressed oral cavity itchiness once at the beginning of SLIT. Patient 2 had no adverse reaction. The levels of antigen-specific IgE decreased in both patients after SLIT, and further decreased after switching to OIT.

**Conclusions:**

Few clinical studies have evaluated the efficacy and safety of SLIT for egg allergy. Although the treatment was conducted in only two patients, our results have shown that SLIT is a promising treatment procedure for egg allergy. Further clinical trials will be needed to additionally assess the efficacy and safety of SLIT in children with food allergy.

## Background

Egg allergy is one of the most common immunoglobulin E (IgE) mediated food allergies in children [1]. Although eliminating the causative food allergen from the child’s diet is the current standard of care, this creates a tremendous burden on their families. In addition, complete adherence to egg-free diet is difficult because a variety of food contain hidden or difficult to recognize egg ingredients such as ovalbumin and ovomucoid. As a result, children with egg allergy constantly have a risk of anaphylaxis resulting from accidental ingestion of egg-containing foods.

Recently, oral immunotherapy (OIT) has been considered a promising treatment option for egg allergy. For example, a systematic review of 10 randomized controlled trials has shown that nearly half of children receiving OIT were able to tolerate a full serving of egg [2]. However, this approach has safety issues with 8.4% of patients receiving OIT having severe adverse reactions requiring epinephrine [2]. Hence, establishing a safer method to treat egg allergy is advisable.

Sublingual immunotherapy (SLIT) is another treatment option, in which an allergen extract is held under the tongue for a short period of time. Mechanistically, SLIT may have some advantages by delivering the allergen to the antigen-presenting cells in oral mucosa. However, few clinical studies have evaluated its efficacy and safety. Recently, we treated two children with severe egg allergy with SLIT. Here, we report these cases.

## Case presentation

Patient 1 was a 7-year-old girl with a history of Group B streptococcus meningitis before 1 year of age. Because she had atopic dermatitis, a blood test for antigen-specific IgE was performed at 10 months of age, resulting 90.7 UA/mL and 48.2 UA/mL for egg-white and ovomucoid respectively. Since then, egg and also peanut (which she also had an elevation of specific antibodies) had been eliminated from her diet. At 2 years of age, accidental consumption of egg white powder contained in a salad lead to eruption of urticaria and vomiting. When she was 3 years old, OFC was performed with a total consumption of 0.3 g of whole egg leading to cough symptoms. By the time of intervention with SLIT, she also had been diagnosed as asthma and allergic rhinitis complementing with egg allergy.

Patient 2 was a 5-year-old girl who had a history of anaphylaxis at 10 months of age when she took a small amount of boiled egg yolk. Furthermore, she had mild asthma and food allergy for salmon roe.

Although OIT for egg was attempted in both patients, anaphylactic symptoms were induced by ingesting only 0.1 g of heated whole egg as a single dose (vomiting, abdominal discomfort, urticaria for patient 1 and nausea, yawning, throat discomfort, decrease of blood pressure for patient 2). Because the patients and their parents were eager to receive treatment for egg allergy, we decided to attempt SLIT. The treatment was conducted after achieving an approval from the institutional review board of the Jikei University School of Medicine. Written informed consent was obtained from the parents.

SLIT was conducted in accordance with the following procedures. Aqueous suspensions were prepared by adding water to the heated whole egg powder (containing an equivalent of 0.69 g egg in 1.2 mL≒120 mg of egg protein). Each suspension was stored in a frozen state and thawed before usage. The solution was administered sublingually, kept in the mouth for 2 min, and spat out immediately thereafter.

For Patient 1, the dose of SLIT was increased gradually from the starting dose of 0.04 g to 0.69 g under the hospitalized setting for 4 days (Table 1). For Patient 2, the starting dose was 0.04 g, and the dose was increased to 0.08 g in the hospital on Day 1. Thereafter, SLIT was conducted at home. The dose was increased again in the hospital on Days 8, 36, and 64 (Table 1). We instructed the parents of both patients to inject epinephrine (Epipen^®^, Mylan N. V., Tokyo) in case anaphylactic symptoms were observed at home.

**Table 1:** Dose titration schedule of sublingual immunotherapy

	Dose equivalent to the whole egg (g)				
Patient 1					
Day 1^a^	0.04	0.07	0.15	0.22	
Day 2^a^	0.11	0.17	0.23	0.34	0.46
Day 3^a^	0.34	0.46	0.57	0.69	
Day 4^a^	0.69	0.69	0.69		
After Day 5	0.69				
Patient 2					
Day 1^a^	0.04	0.08			
Days 2 to 7	0.08				
Day 8^a^	0.16	0.24			
Days 9 to 35	0.24				
Day 36^a^	0.3	0.4			
Days 37 to 64	0.4				
Day 65^a^	0.5	0.6			
After Day 65	0.6				

^a^Dose was increased at 2- or 3-h intervals in the hospital

Once the dose reached 0.69 g for Patient 1 and 0.6 g for Patient 2, both patients continued daily maintenance SLIT at home. At first, SLIT was scheduled for 6 months. However, it was continued for 7 months for Patient 1 and 8 months for Patient 2 due to the exploratory character of the study. During the treatment, Patient 1 reported itchiness of the oral cavity just once when the dose was increased to 0.34 g on Day 2. No adverse reaction was observed in Patient 2. The level of antigen-specific IgE decreased in both patients after SLIT (Table 2).

**Table 2:** Changes in the antigen-specific immunoglobulin E levels

	Patient 1	Patient 2
Egg white specific IgE (uA/mL)		
Before starting SLIT	56.1	14.4
At the completion of SLIT	42.8	9.65
During OIT (at Mar/2020)	18.7	6.29
Ovomucoid specific IgE (uA/mL)		
Before starting SLIT	37.7	10.3
At the completion of SLIT	33.8	6.79
During OIT (at Mar/2020)	12.4	4.59

*IgE* immunoglobulin E, *SLIT *Sublingual immunotherapy, and *OIT* oral immunotherapy

Because the maximum liquid volume of the suspension which could be held in the sublingual space of children was considered to be 1.2 mL, we could no further increase the dose of SLIT. In addition, the long-term efficacy of OIT had been recognized to be superior to that of SLIT [3]. As a result, SLIT was switched to OIT with low-dose heated whole egg, which was conducted by modifying the procedures previously reported [4]. After switching to OIT, the dose was gradually increased by 10% or 20% until achieving a maintenance dose of 1 g of heated whole egg (≒170 mg of egg protein) until June 2020. During OIT, no adverse reaction was observed in both patients, and the antigen-specific IgE levels decreased further (Table 2). Since we have not performed oral challenge tests after discontinuation of OIT, we cannot confirm whether the patients successfully achieved true tolerance or not. However, the fact that these patients are able to take 1 g of heated whole egg safely every day, and the decrease of antigen-specific IgE levels indicates that these patients are now in the process of achieving tolerance. Thus, we are planning to continue OIT for these patients for the next several years.

## Discussion and conclusions

Immunotherapy aims to induce immune tolerance by administering a confirmed allergen and can potentially be used as a curative treatment for food allergy [5]. However, OIT can induce serious adverse reactions such as anaphylaxis [6]. For this reason, OIT is not recommended as a standard treatment in most major treatment guidelines [7]. In contrast, SLIT is expected to minimize severe adverse reactions [8]. It may also have advantages by delivering the antigen to the antigen-presenting cells in an intact form before digestion in the stomach [9].

SLIT has been widely used as a method of treating allergic diseases induced by aeroallergens such as mite antigens or pollen antigens, [10] but few clinical studies have evaluated the efficacy and safety of SLIT for egg allergy. In our investigation, we were able to safely treat two children with SLIT followed by OIT. Initially, these children gave up OIT because of serious allergic reactions even at a low dose of 0.1 g of heated whole egg. Although the treatment was conducted in only two patients, our results suggest that SLIT may be a promising treatment approach for egg allergy. Finally, even though current standard therapeutic options for a motivated family/patient who are unable to tolerate standard OIT would be a combination of omalizumab with typical OIT, [11] SLIT would be a much less expensive and less invasive option for such patients. Further clinical trials will be needed to additionally assess the efficacy and safety of SLIT in children with food allergy.

## Data Availability

All data generated or analyzed during this study are included in this published article.

## Abbreviations

IgE: Immunoglobulin E
SLIT: Sublingual immunotherapy
OIT: Oral immunotherapy
